# Distribution, genetic characteristics and public health implications of *Triatoma rubrofasciata*, the vector of Chagas disease in Guangxi, China

**DOI:** 10.1186/s13071-020-3903-z

**Published:** 2020-01-20

**Authors:** Yunliang Shi, Yaobao Wei, Xiangyang Feng, Jianfeng Liu, Zhihua Jiang, Fangqi Ou, Haiyan Wei, Guoli Lv, Xiaoling Wan, Ziyue Wang, Yichao Yang

**Affiliations:** 1 Institute of Parasitic Disease Prevention and Control, Guangxi Zhuang Autonomous Region Center for Disease Control and Prevention, Nanning, 530028 China; 2 Jiangsu Institute of Parasitic Diseases, Wuxi, 214064 China; 3 Guangxi Medical University, Nanning, 530021 China

**Keywords:** *Triatoma rubrofasciata*, Guangxi, Distribution, Genetic characteristics

## Abstract

**Background:**

Triatomines are natural vectors of Chagas disease and are mainly prevalent in the Americas. In China, previous data from decades ago showed that there were two species of triatomine bugs, *Triatoma rubrofasciata* and *T. sinica*. However, the distribution, genetic characteristics and public health implications of triatomines in China are still relatively unknown. In order to gain knowledge on the distribution, genetic characteristics and public health implications of the triatomines in Guangxi, China, an entomological-epidemiological study and genetic research was conducted.

**Methods:**

Different methods were used to elucidate the distribution of triatomines in Guangxi including consultations with county-level Center for Disease Prevention and Control staff and village doctors, the distribution of educational material on triatomines though the internet and social media apps such as Wechat and QQ, and conducting manual inspections and light trapping to collect triatomines. The morphological characteristics of the collected triatomines were identified under light microscopy. The mitochondrial *16S* rRNA, cytochrome *b* (*cytb*) genes and nuclear *28S* rRNA gene were amplified, sequenced and used in phylogenetic analyses.

**Results:**

A total of 305 triatomines were captured from 54 different sites in 13 cities in Guangxi. All collected bugs were identified as *T. rubrofasciata* based on morphology. Most triatomine collection sites were around or inside houses. Four triatomines bite cases were observed during the investigation indicating that triatomine bites are common, the bites can cause serious anaphylaxis and skin papules and urticaria, suggesting a systemic skin response. The *16S* rRNA, *28S* rRNA and *cytb* sequence analyses of *T. rubrofasciata* from Guangxi and other countries showed that *T. rubrofasciata* sequences from different regions exhibit a high similarity, with no geographical differences. The phylogenetic tree based on the *16S* rRNA and *cytb* genes showed that *T. rubrofasciata* sequences from different regions and continents were in the same cluster, indicating no differentiation among different geographical populations.

**Conclusions:**

Our study showed that *T. rubrofasciata* is widely distributed in Guangxi and that people are commonly bitten by this insect in some regions. This highlights the need to enhance surveillance for and control of *T. rubrofasciata* and to strengthen the monitoring of imported *Trypanosoma cruzi* in China. The *16S* rRNA, *28S* rRNA and *cytb* sequence analyses of *T. rubrofasciata* from different regions and continents suggested that *T. rubrofasciata* populations exhibit high similarity, and the clustering in the phylogenetic analyses indicates that *T. rubrofasciata* has a close ancestor originating in the Americas.

## Background

Chagas disease, or American trypanosomiasis, is one of the 10 most seriously neglected tropical diseases [1] and currently affects 10 million people worldwide [2]. There is no vaccine or effective cure once the symptoms of the chronic disease have manifested. Chagas disease is considered limited to the Americas; however, in recent decades, it has become a global health issue [3]. Due to the growing rate of immigrants unaware of their own infection status [4], the disease can potentially spread to non-endemic countries. Moreover, *Trypanosoma cruzi* Chagas, 1909 (Kinetoplastida, Trypanosomatidae) can be transmitted by blood transfusion and organ transplantation. With increasing transcontinental exchanges, Chagas disease is no longer restricted to the Americas [5, 6]. Currently, it has been diagnosed in several non-endemic countries, such as New Zealand, Australia, Japan and Europe [7–9].

Chagas disease is transmitted mainly by triatomine bugs (kissing bugs) [10] belonging to 18 genera and 154 species worldwide [11–16]. *Triatoma rubrofasciata* (De Geer, 1773) is the only species with a worldwide distribution [17]. It is mostly found in Asia, Oceania, Africa and Central America. It is a common vector of *Trypanosoma conorhini* (Donovan) that infects *Rattus rattus* [18, 19]. *T. rubrofasciata* is also occasionally found naturally infected by *T. cruzi* [20, 21]. Additionally, *T. rubrofasciata* bites can cause dermatitis, anaphylactic shock, and even death [22, 23].

In China, very few records have indicated that *T. rubrofasciata* exists in southern China, such as in Guangxi Zhuang Autonomous Region, Guangdong and Fujian provinces [24–26]. Furthermore, the peridomestic presence of *T. rubrofasciata* was reported to the northeast of Hanoi, extending across the frontier well into southern China [27]. However, there have been few studies on *T. rubrofasciata* over the decades in China, and a very limited amount of information about its distribution has been published. Two blood-sucking triatomines, *T. rubrofasciata* and *T. sinica* Hsiao, 1965, have been reported in China over the past five decades [24]. Nine cases of *T. rubrofasciata* invading houses and biting people, resulting in anaphylactic shock or death, have been reported in China [23, 28, 29]. However, blood-sucking triatomines have received limited attention for decades. People are not sufficiently concerned about this insect or aware of the consequences. Several publications ranging from the characterization of the number of chromosomes [30] to the description of the genome [31] and its phylogenetic relationships [32] have contributed to the genetic, taxonomic and evolutionary knowledge of *T. rubrofasciata*. However, little is known about the actual distribution of this vector in China. Thus, we conducted an entomological-epidemiological study and genetic research in Guangxi, China.

## Methods

### Triatomines and triatomine bite investigations

Triatomine collection in the field was carried out from July 2016 to October 2018 in Guangxi, China. First, we asked the county level Center for Disease Prevention and Control staff and village doctors about the presence of *T. rubrofasciata* in its suspected distribution areas and showed them pictures of triatomines. The fieldwork involved inspection of old houses (houses made of clay), tree cavities, wood and rock piles, chicken coops and dog kennels. Additionally, light traps were used to collect triatomines. The light installation followed the method by Castro et al. [33] and included a mobile electric power pack and approximately a 1.8 × 2.2 m vertical aluminium frame with a white sheet. The frame was kept erect with guide ropes. On both sides of the frame, two 125 W and 250 W mercury vapor bulbs (HPL) were suspended with external ballasts. The light trap was run from sunset until midnight and the insects on the sheet were checked every hour. Geographical coordinates of the location from where the specimens were caught were recorded with a handheld GPS unit (UniStrong, Beijing, China) for each site. To obtain more information on triatomine distribution, we disseminated electronic posters and leaflets about the triatomine bugs on the internet and social media Apps such as Wechat. The electronic poster and leaflets were also distributed to the staff of the County and City Disease Prevention and Control units. People who found the bugs sent photos with locations. We identified the species of the triatomine based on the photos and went to the location to collect the specimen. In the locations where triatomines were found, we interviewed the local people about how often they get bitten by triatomines. Information from each individual who had been bitten by triatomines was collected, including personal information, symptomatic descriptions, treatment and follow-up visits.

### DNA extraction, amplification and sequencing

From 13 collected triatomines, genomic DNA was extracted from two legs of each of the triatomines using the QIAamp DNA Mini Kit (Qiagen, Hilden, Germany) according to the manufacturer’s recommendations. The mitochondrial *16S* rRNA and *cytb* gene and the nuclear ribosomal *28S* rRNA gene were PCR-amplified using established primers (Table 1). PCR was conducted in a final volume of 25 μl containing 2× Taq PCR Master Mix (Takara, Dalian, China), 0.4 μM of each primer and 2 μl DNA template. The following PCR cycling conditions were employed: 95 °C for 3 min; 35 cycles of 94 °C for 45 s, 55 °C for 45 s, and 72 °C for 1 min; and a final extension step at 72 °C for 10 min. PCR products were analysed by electrophoresis in a 1.5% agarose gel. Amplified DNA was purified from the gel using the Agarose Gel DNA Extraction Kit (Takara), and the DNA was sequenced at Sangon Biotech (Shanghai, China).

**Table 1:** The primers used for *16S* rRNA, *28S* rRNA and *cytb* sequence amplification

Marker	Forward primer (5’–3’)	Reverse primer (5’-3’)	Amplicon length (bp)
*16S* rRNA	CGCCTGTTTATCAAAAACAT	CTCCGGTTTGAACTCAGATCA [34]	250
*cytb*	GGACGWGGWATTTATTATGGATC	GCWCCAATTCARGTTARTAA [32]	250
*28S* rRNA	GCGAGTCGTGTTGCTTGATAGTGCAG	TTGGTCCGTGTTTCAAGACGGG [35]	300

### Sequence alignment and phylogenetic analyses

The sequences were aligned by ClustalW (https://www.genome.jp/tools-bin/clustalw) and any mutations were detected. The sequences were compared with those available in the GenBank database using the Basic Local Alignment Search Tool (BLASTn http://blast.ncbi.nlm.nih.gov/Blast.cgi). The phylogenetic tree for triatomines from Guangxi, along with other homologous sequences (rubrofasciata clade) for the *16S* rRNA and *cytb* genes were constructed using the neighbour-joining (NJ) method with 1000 bootstrap replications using MEGA7.0 [36].

## Results

### The distribution of triatomines in Guangxi

**Fig. 1:**
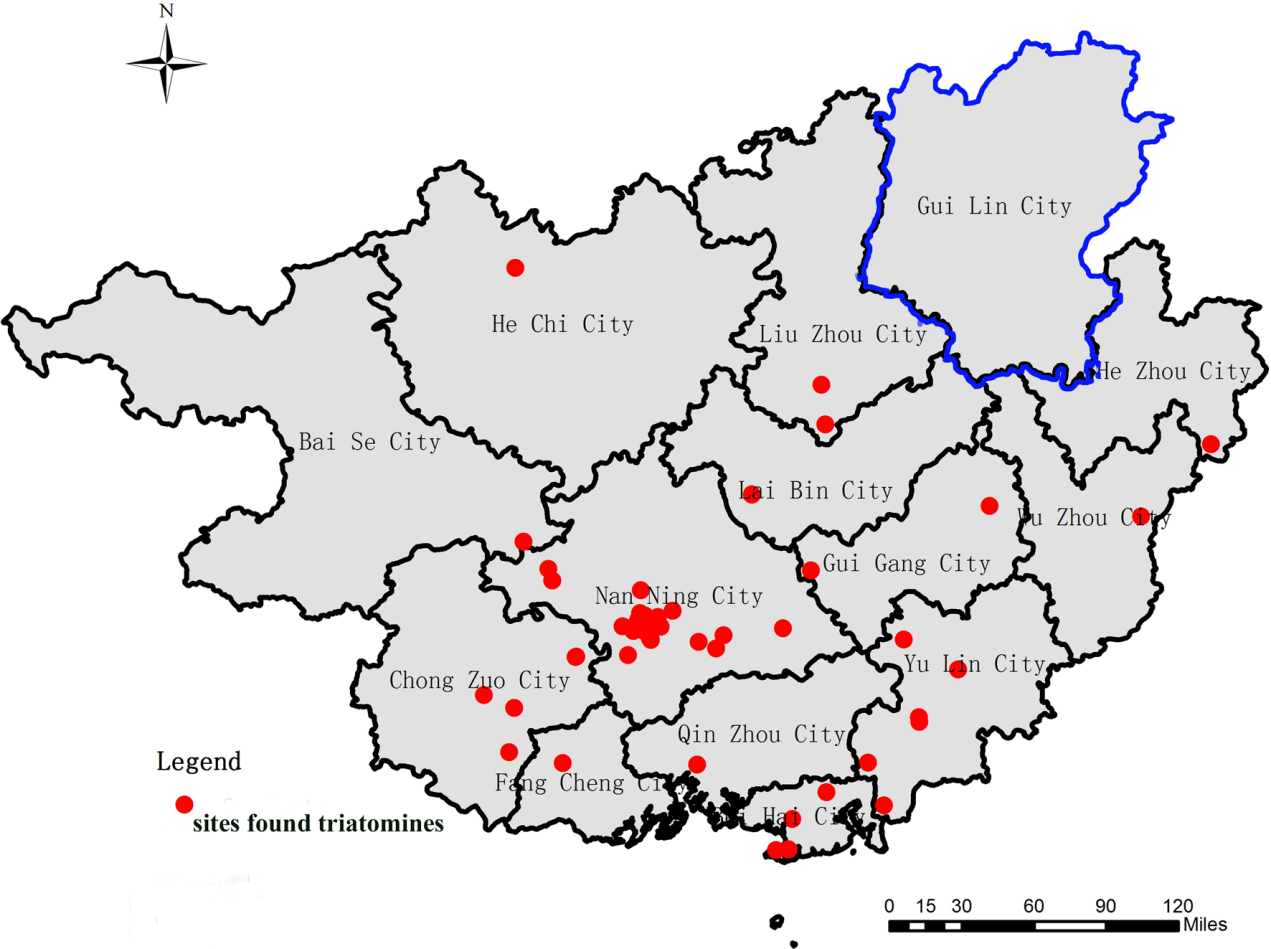
The distribution of *Triatoma rubrofasciata* in Guangxi, China. Over the two years of study, triatomines were found in 54 cities (red dots). Except for Guilin city (highlighted with blue), 13 other cities in Guangxi Zhuang Autonomous Region were positive for *T. rubrofasciata*

**Fig. 2:**
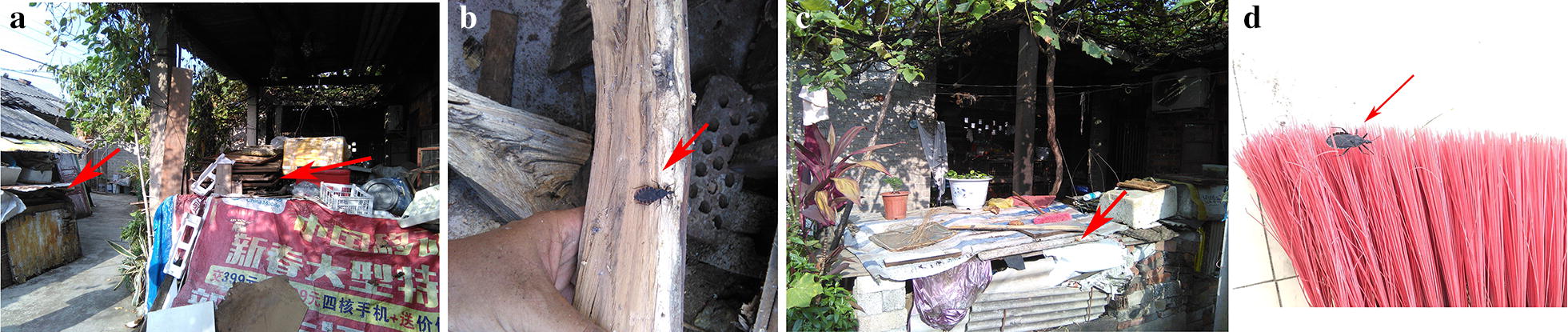
The living habit of *Triatoma rubrofasciata.* **a** *T. rubrofasciata* tend to hide in woodpiles; **b** *T. rubrofasciata* found in a woodpile; **c** *T. rubrofasciata* tend to hide near chicken coops. **d** *T. rubrofasciata* found in a house when cleaning

According to the field investigation results and feedback from the internet and social media apps, 54 sites across 13 different cities in Guangxi Zhuang Autonomous Region were positive for blood-sucking triatomines (Fig. 1). The most common places where triatomines were found were inside or around houses (Fig. 2a–d), such as in wood piles (Fig. 2a, b) and chicken coops (Fig. 2c), in both urban and rural locations. A total of 305 triatomines were collected, including juvenile and adult bugs (see Additional file 1: Table S1 for additional information for collected triatomines). A total of 42.62% (*n* = 130) of the specimens were female, 40% (*n* = 122) were male, 17.38% (*n* = 53) were nymphs, and 31.15% (*n* = 95) had fed on blood. The number of collected insects at a single location was very different, ranging from one to over one hundred.

### Morphology of the triatomines

**Fig. 3:**
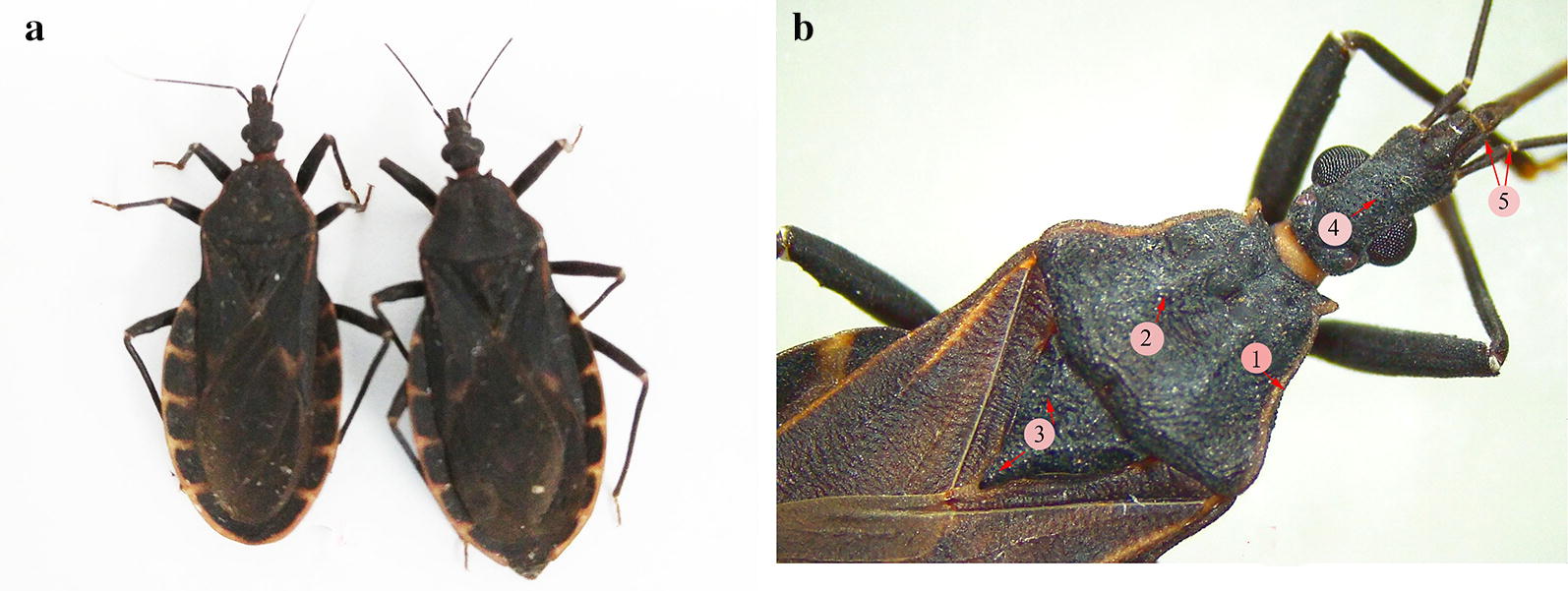
Morphology of *Triatoma rubrofasciata.* **a** An adult male (left) and female (right). **b** Morphological characteristics of *T. rubrofasciata*: 1, there is an orange-red margin along the outer edge of the abdomen as well as the side of the pronotum; 2, the pronotum is dark brown or black and conspicuously granulose; 3, the scutellum is wide at the base and tapers to the tip; 4, the head is uniformly dark and heavily granulose dorsally; 5, the 1st segment of antenna surpasses the head

The collected juvenile and adult triatomine bugs were identified as *T. rubrofasciata* by morphological characteristics following Xiao et al. [24] and the website for *T*. *rubrofasciata* of the Centers for Disease Control and Prevention USA (https://www.cdc.gov/parasites/chagas/gen_info/vectors/t_rubrofasciata.html). The length of collected fourth-instar larvae was approximately 10 mm. Adult *T. rubrofasciata* morphology showed that the length of male bugs was 19.0–24.0 mm, and the length of females was 20–25 mm (Fig. 3a). The overall colour was dark brown to black, and its entire outer margin was delicately bordered with orange-red, with yellowish and orange-red markings on the neck, pronotum, corium and connexium. The dorsal surface was conspicuously granulose, while the head and pronotum were heavily granulose dorsally. The scutellum had the general body colour and was rugose-granulose. The posterior process was conical, subtriangular, and strongly tapered from a wide base to a point. The head was uniformly dark, with the first antennal segment distinctly projecting beyond the head (Fig. 3b).

### People bitten by *T. rubrofasciata*

**Fig. 4:**
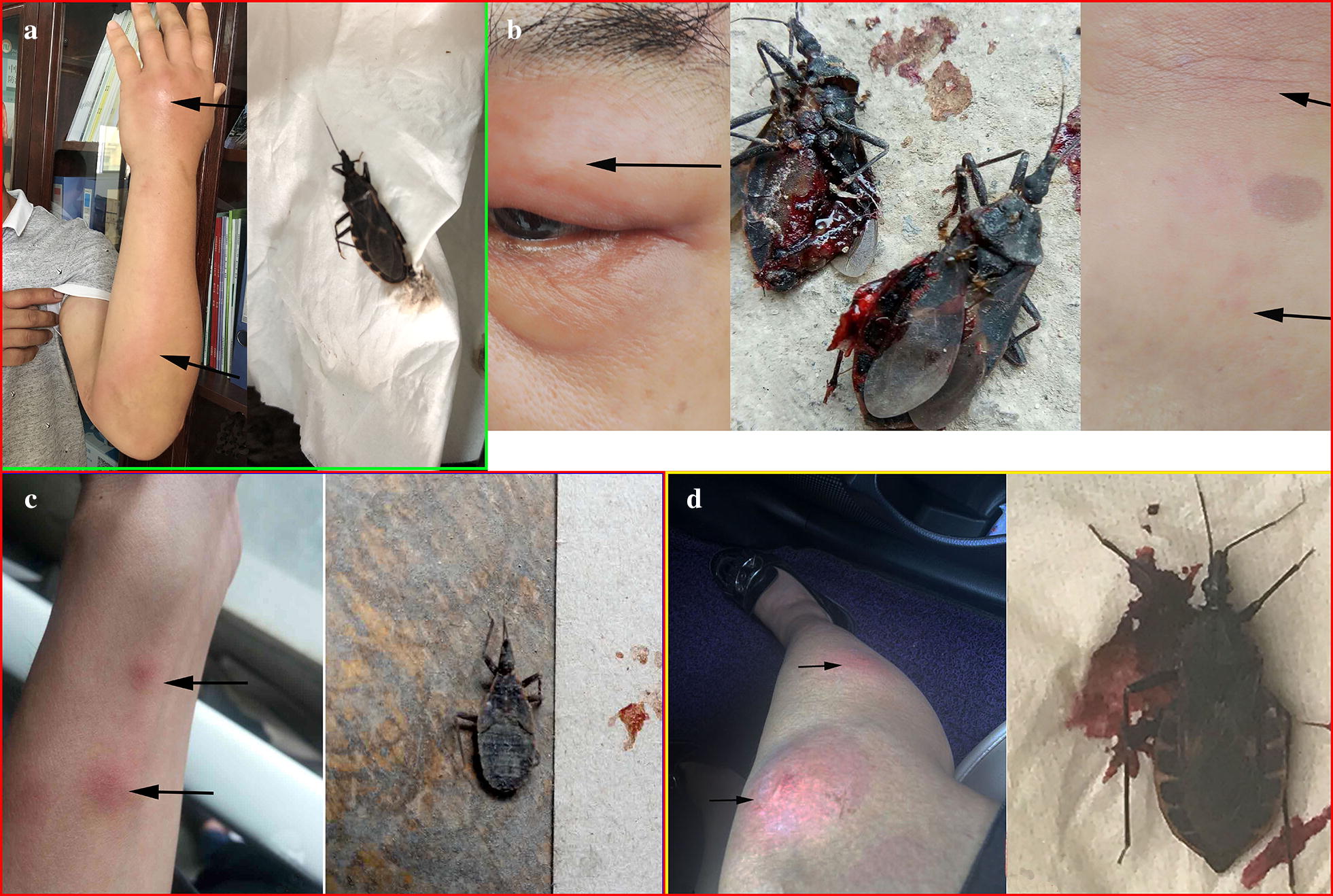
Four *Triatoma rubrofasciata* bite cases. **a** A bite by an adult *T. rubrofasciata* caused cutaneous symptoms including urticaria, flushing, pruritus, and angioedema. **b** A bite by an adult *T. rubrofasciata* (middle) caused palpebral oedema (left) and slight skin redness on the back (right). **c** A bite by a fifth-instar *T. rubrofasciata* (left) caused an urticarial skin reaction and slight redness on the arm (right). **d** A woman was bitten by an adult *T. rubrofasciata* on her foot, thigh and knees while sleeping

Our inquiry showed that over 20 people had been bitten by *T. rubrofasciata* in the past year, and over one hundred people said that they had experienced a bite by *T. rubrofasciata.* Because many of the people bitten by *T. rubrofasciata* did not show serious clinical signs and recovered within approximately two weeks, they did not pay attention to the bite. Four cases of *T. rubrofasciata* bites were observed during the investigation (Fig. 4a–d). The ages of the patients were 26, 30, 35 and 52 years-old. All of them were bitten during noon break or night sleep in their living quarters. The bite sites included the back of the hand, forearm, eyelid, shin, thigh and back. Bites can cause serious anaphylactic reactions, including skin bullae, vesicles, papules, surrounding swelling or erythema and urticaria, suggesting a systemic skin response. One patient presented generalized cutaneous symptoms, including urticaria, flushing, pruritus, and angioedema (Fig. 4a); another patient experienced a headache and muscle aches (Fig. 4c). Urticaria and other clinical signs tend to disappear within approximately two weeks. The triatomines were caught after biting and were identified as *T. rubrofasciata* (Fig. 4).

### Genetic analysis and genetic relatedness

**Fig. 5:**
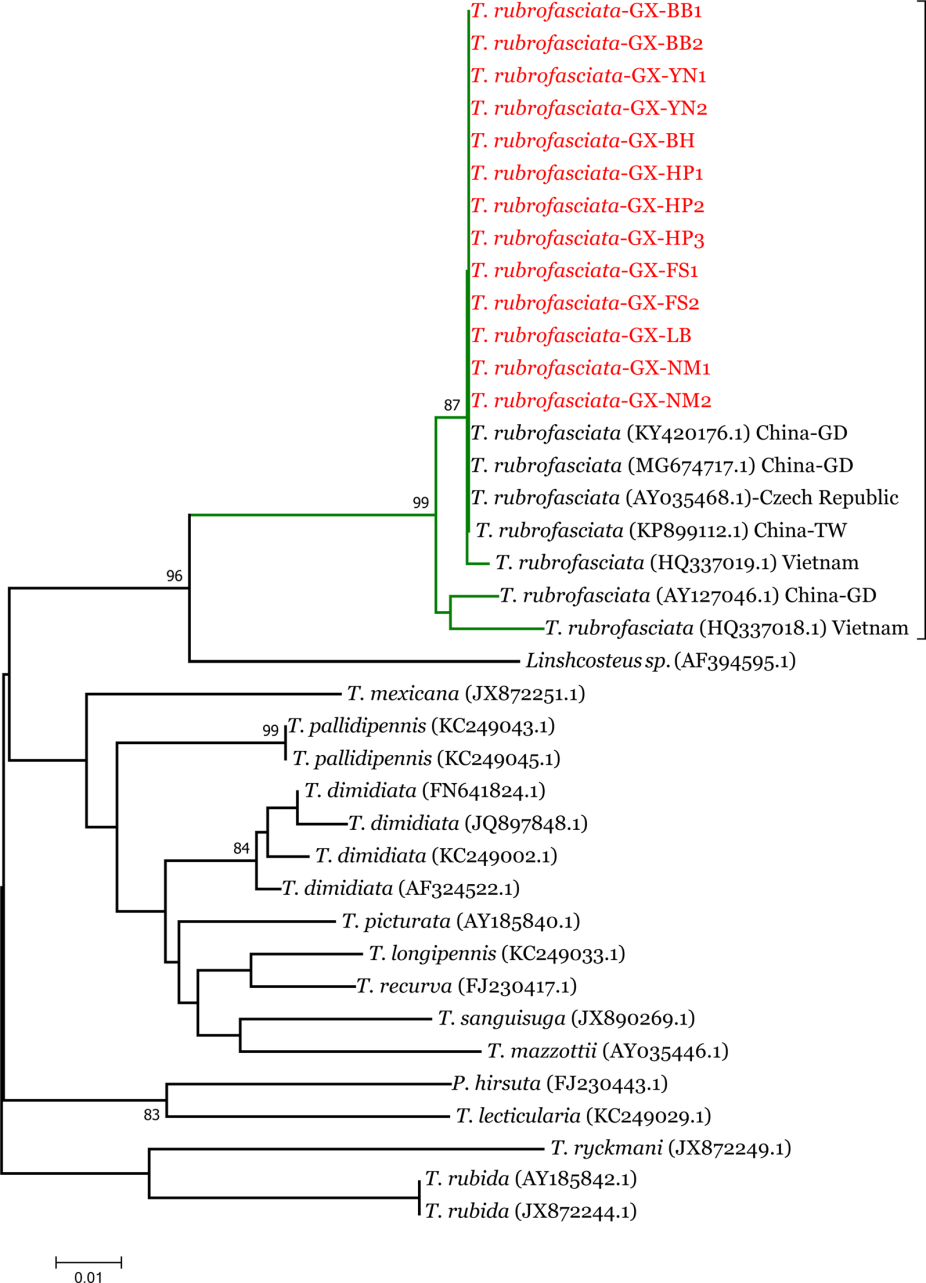
The phylogenetic tree based on the *16S* rRNA gene sequences for *Triatoma rubrofasciata* from Guangxi and other related species. The phylogenetic tree constructed by MEGA using the neighbour-joining (NJ) method with 1000 bootstrap replications. The sequences from this study were highlighted with red color. *Abbreviations*: GD, Guangdong Province; TW, Taiwan

The 13 triatomines collected were from the Haicheng district (*n* = 1) and Hepu County (*n* = 3) of Beihai city; Bobai County (*n* = 2) of Yulin city; Yongning County (*n* = 2) of Nanning city; and Fusui County (*n* = 2), Ningming County (*n* = 2) and Luobai town (*n* = 1) of Chongzuo city. The DNA was amplified, and the mitochondrial (*16S* rRNA and *cytb*) and nuclear (*28S* rRNA) genes were sequenced. Multiple 499-bp fragments of the *16S* rRNA gene from *T. rubrofasciata* were obtained, and the sequences were submitted to the GenBank database under the accession numbers MH236899.1–MH236905.1. The NCBI BLAST search indicated that the isolated triatomine bugs had over 98% identity with *T. rubrofasciata* from China (GenBank: MG674717.1, KY420176.1, KP899112.1 and AY127046.1), the Czech Republic (GenBank: AY035468.1) and Vietnam (GenBank: HQ337019.1 and HQ337018.1). The *16S* rRNA gene sequences from 13 *T. rubrofasciata* from Guangxi (Infomation see Table 2) that were aligned with the sequences of *T. rubrofasciata* from Vietnam, the Czech Republic and other places in China showed nine polymorphic sites; seven mutations were found in a single sequence, and the other two mutated sites were detected in multiple sequences (Additional file 2: Figure S1). The phylogenetic tree based on the *16S* rRNA gene showed that all *T. rubrofasciata* from China, Vietnam and the Czech Republic were in the same cluster (Fig. 5), with a bootstrap value of 99. The cluster could be divided into two separate branches; the first branch contained *T. rubrofasciata* from Guangdong Province (GenBank: AY127046.1) and Vietnam (GenBank: HQ337018.1). The other branch contained the sequences from China, Vietnam and the Czech Republic. The phylogenetic relationships of *T. rubrofasciata* populations were not associated with their geographical distribution. *Triatoma rubrofasciata* is closely related to *Linshcosteus* spp. (GenBank: AF394595.1) from India, the only triatomine genus exclusively from the Old World (Fig. 5).

**Table 2:** Information of *Triatoma rubrofasciata* isolates from Guangxi used in the *16S* rRNA and *cytb* sequence phylogenetic analyses

Isolate	Locality	Latitude/Longitude	Date	GenBank ID
BB-1	Yunlin City, Bobai County	22°27ʹ28.95ʺN, 109°96ʹ99.93ʺE	2017	MH236899.1 (*16S*); MH368015.1 (*cytb*)
BB-2	Yunlin City, Bobai County	21°74ʹ30.31ʺN, 109°75ʹ84.17ʺE	2017	
YN-1	Nanning City, Yongning County	22°68ʹ68.38ʺN, 108°74ʹ86.31ʺE	2017	MH236900.1 (*16S*); MH368016.1 (*cytb*)
YN-2	Nanning City, Yongning County	22°68ʹ68.38ʺN, 108°.74ʹ86.31ʺE	2017	
FS-1	Chongzuo City, Fusui County	22°63ʹ64.84ʺN, 107°90ʹ89.09ʺE	2017	MH236903.1 (*16S*); MH368020.1 (*cytb*)
FS-2	Chongzuo City, Fusui County	22°63ʹ49.75ʺN, 107°90ʹ41.06ʺE	2017	
LB	Chongzuo City, Jiangzhou District	22°40ʹ52.00ʺN, 107°35ʹ19.25ʺE	2017	MH236904.1 (*16S*); MH368019.1 (*cytb*)
NM-1	Chongzuo City, Ningming County	22°06ʹ27.24ʺN, 107°50ʹ35.50ʺE	2017	MH236905.1 (*16S*); MH368021.1 (cytb)
NM-2	Chongzuo City, Ningming County	22°06ʹ27.24ʺN, 107°50ʹ35.50ʺE	2017	
BH	Beihai City, Haicheng District	21°47ʹ88.52ʺN, 109°18ʹ45.86ʺE	2017	MH236901.1 (*16S*); MH368017.1 (*cytb*)
HP-1	Beihai City, Hepu County	21°82ʹ13.00ʺN, 109°41ʹ40.26ʺE	2017	MH236902.1 (*16S*); MH368018.1 (*cytb*)
HP-2	Beihai City, Hepu County	21°82ʹ13.00ʺN, 109°41ʹ40.26ʺE	2017	
HP-3	Beihai City, Hepu County	21°66ʹ09.00ʺN, 109°20ʹ725.11ʺE	2017

A 666-bp fragment of the *28S* rRNA gene from the collected *T. rubrofasciata* was amplified. The sequences were submitted to the GenBank database under the accession numbers MH356275.1-MH356281.1. The BLAST search indicated that the isolated triatomines had the greatest homology (over 99%) with *T. rubrofasciata* from China (GenBank: MG675575.1 and KY420177.1), Brazil (GenBank: KR632546.1), Vietnam (GenBank: KR632547.1 and KR632548.1) and the USA (GenBank: GQ853371.1). The alignment of the *T. rubrofasciata 28S* rDNA sequences from GenBank showed six mutations in a single sequence among the *T. rubrofasciata* from China, Uruguay and the USA (Additional file 2: Figure S1).

**Fig. 6:**
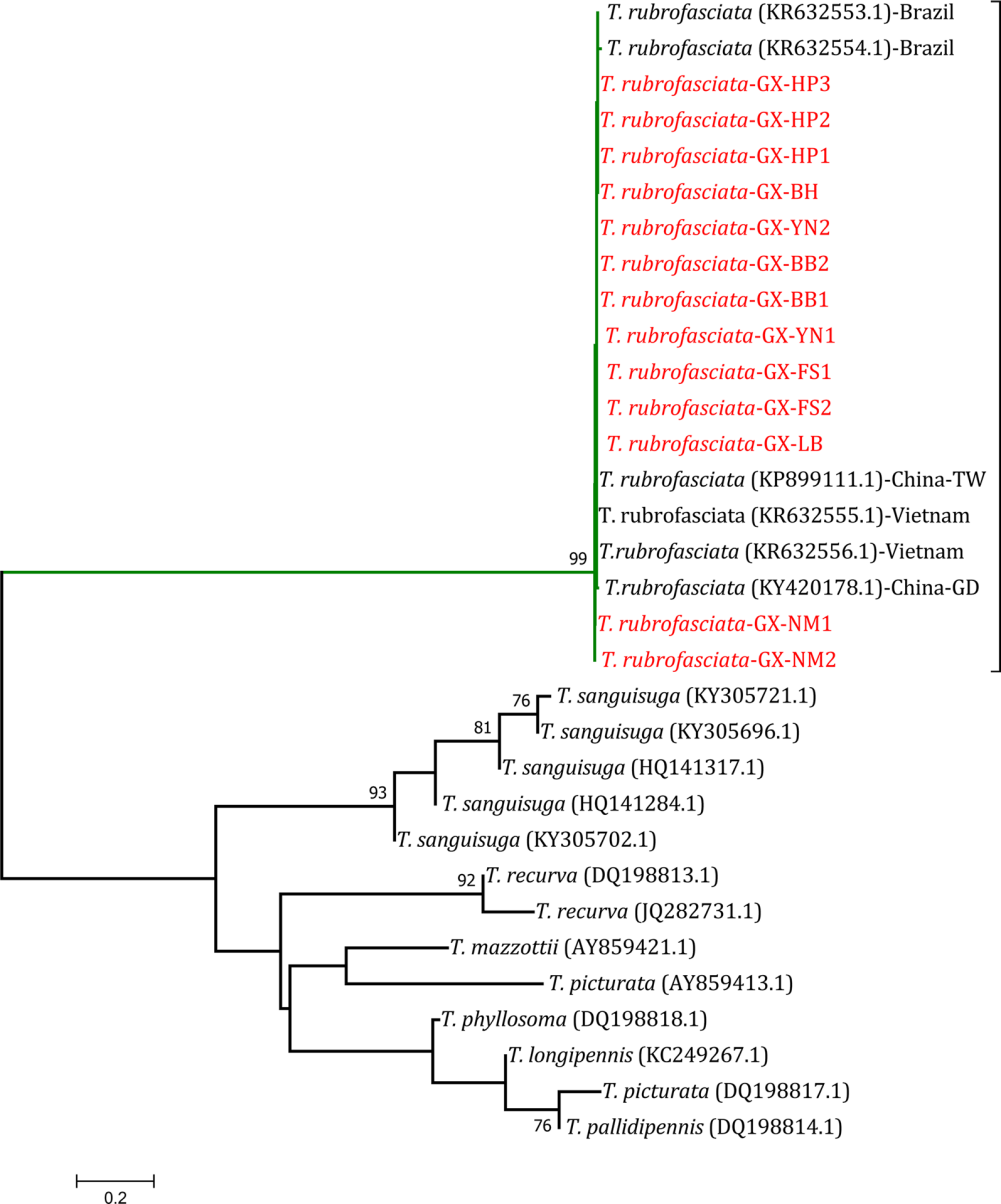
The phylogenetic tree based on the *cytb* gene sequences for *Triatoma rubrofasciata* from Guangxi and other related species. The phylogenetic tree constructed by MEGA using the neighbour-joining (NJ) method with 1000 bootstrap replications. The sequences from this study were highlighted with red color. *Abbreviations*: GD, Guangdong Province; TW, Taiwan

A 667-bp fragment of the *cytb* gene from the collected *T. rubrofasciata* was amplified, and the sequences were submitted to the GenBank database under the accession numbers MH368015.1-MH368021.1. The BLAST search indicated that the triatomines had 99% homology with *T. rubrofasciata* from China (GenBank: KP899111.1 and KY420178.1), Brazil (GenBank: KR632554.1 and KR632553.1) and Vietnam (GenBank: KR632555.1 and KR632556.1). Ten mutated sites were found after the alignment of the *cytb* gene of *T. rubrofasciata* from China, Vietnam and Brazil. Four mutations were found in a single sequence, and six other mutations were identified (Additional file 2: Figure S1). The phylogenetic tree showed that all of the *T. rubrofasciata* from China, Brazil and Vietnam were in the same cluster (Fig. 6).

## Discussion

In China, only few studies on *T. rubrofasciata* have been carried out over the past fifty years [24–26], with only a limited amount of information regarding its distribution. To date, the knowledge of the distribution of triatomines in China is still scarce. In the present study, we investigated the distribution of *T. rubrofasciata* in Guangxi using multiple methods, including network reporting, field interviews and field trapping. We collected 305 *T. rubrofasciata*, including nymphs and adults, from 54 different sites across 13 cities in Guangxi. Our study showed that *T. rubrofasciata*, the vector of *T. cruzi*, exists extensively in Guangxi, South China. With increasing intercontinental activities, there is an increasing risk of imported Chagas disease. The wide distribution of *T. rubrofasciata* could support *T. cruzi* colonization, transmission and invasion in new areas.

In this study, we used various methods to survey for *T. rubrofasciata,* including manual inspection, light trapping, internet tools and social media apps such as Wechat and QQ. Among the three methods, light trapping was the least effective, as it did not capture any *T. rubrofasciata*. This could be because of the low distribution density of these kissing bugs. The internet and social media app feedback enabled us to identify most of the *T. rubrofasciata* distribution locations. While the use of a mobile phone is very common in Guangxi (over 90% of people are actively online) Wechat and QQ were very effective tools to communicate and receive insect information in real time; thus, Internet could be powerful tool for disease surveillance and management [37–39]. Since the triatomines had a relatively low population density and because they are active at night, manual inspections were difficult to conduct. The feedback by witnesses and reports by local people was particularly important to locate *T. rubrofasciata*; these reports greatly benefited the manual inspections and insect collections.

In the present study, most of the *T. rubrofasciata* were collected in houses or near living areas, indicating that this insect lives near human habitation and that humans are easily exposed. Our interviews also revealed that people being bitten by *T. rubrofasciata* is common in some regions in Guangxi. Four cases of *T. rubrofasciata* bites were observed during the investigation. Common clinical symptoms are skin papules and an urticaria-like systemic skin response; people will usually recover in approximately one to two weeks. Because most of the people bitten by *T. rubrofasciata* did not show serious clinical symptoms, people did not pay attention to this insect and its bite. However, the prevalence of *T. rubrofasciata* and the common biting activity of *T. rubrofasciata* in Guangxi Zhuang Autonomous Region could make the colonization and spread of *T. cruzi* possible. Moreover, triatomines, as a potential vector of other viruses, bacteria and parasites, can transmit pathogens between humans and animals [40]. Triatomines have a wide range of host blood sources. The pathogens carried by the bug can be stored and propagated in its midgut or maintained in the crop and spread to the salivary glands. Therefore, saliva and faeces are two common ways to spread pathogens to humans and animals [40]. A pathogen in the saliva can enter a hostʼs blood vessels and subcutaneous tissue through a bite. Studies have confirmed that *Trypanosoma rangeli* and *Bartonella* spp. carried by an American blood-sucking triatomine can be transmitted to humans [41–43]. The pathogen-containing faeces excreted by the triatomines can enter the human body through a skin wound or the mucosa, causing infection. Studies have shown that *T. cruzi*, *Serratia marcescens*, *Mycobacterium leprae*, HIV and HBV can be transmitted to humans through triatomine faeces [44–49]. In China, there have been no reports about the infectious diseases transmitted by *T. rubrofasciata*; future research should focus on the acquisition and transmission of pathogens by this insect.

The Triatominae is a subfamily of the Reduviidae that is mainly distributed in the Americas. The current consensus is that the Triatominae have relatively recent origins in the Americas and that the Old World species represent derivatives of an American form [33]. *Triatoma rubrofasciata* is the only species of the Triatominae with a worldwide distribution, and the mode of natural dispersal of *T. rubrofasciata* to other areas and to the Old World is unknown [34]. One hypothesis is that mice infested with *T. rubrofasciata* were accidentally carried to the Old World and other regions on ships, resulting in the current worldwide dispersal of *T. rubrofasciata* [50, 51]. The other hypothesis is that the Bering Land Bridge in the mid-Oligocene was a route for terrestrial plants and animals, as well as triatomines, that migrated between North America and Asia [51]; perhaps the land bridge provided suitable conditions to facilitate the dispersal of triatomines. In the present study, the sequence alignment of *T. rubrofasciata* from different regions and continents exhibited a high similarity and phylogenetic analyses showed they were in the same cluster, indicating that *T. rubrofasciata* has a close ancestor originating in the Americas.

## Conclusions

Our study showed that *T. rubrofasciata* is widely distributed in Guangxi Zhuang Autonomous Region, southern China, and in some regions, people are commonly bitten by this insect. This highlights the need to enhance surveillance and control of *T. rubrofasciata* and strengthen the monitoring of imported *T. cruzi* in China. The *16S* rRNA, *28S* rRNA and *cytb* sequence analyses of *T. rubrofasciata* from different regions and continents suggested that *T. rubrofasciata* populations exhibited high similarity, and clustered in the same cluster in the phylogenetic analyses, indicating that *T. rubrofasciata* has a close ancestor originating in the Americas.

## Supplementary information

**Additional file 1: Table S1.** Data for *T. rubrofasciata* collected in Guangxi, China.

**Additional file 2: Figure S1.** Alignments of *16S* rRNA, *28S* rRNA and *cytb* genes of *T. rubrofasciata* from Guangxi, China.

## Data Availability

The newly generated sequences were submitted to the GenBank database under the accession numbers MH236899.1–MH236905.1 (*16S* rRNA gene), MH356275.1-MH356281.1 (*28S* rRNA gene) and MH368015.1-MH368021.1 (*cytb*). The data from this study are available from the corresponding author upon request.

## Abbreviations

*cytb*: cytochrome b
NJ: neighbour-joining
