# Supplementary material for: Distribution, genetic characteristics and public health implications of Triatoma rubrofasciata, the vector of Chagas disease in Guangxi, China
Source: Parasit Vectors. 2020 Jan 20;13:33. doi: 10.1186/s13071-020-3903-z (PMC6972020; doi:10.1186/s13071-020-3903-z)
Supplement: Supplementary file 2 — Additional file 2: Figure S1. Alignments of 16S rRNA, 28S rRNA and cytb genes of T. rubrofasciata from Guangxi, China. [file 13071_2020_3903_MOESM2_ESM.pdf]

The alignment of 16S rRNA gene of *Triatoma rubrofasciata* from Guangxi

```
16BB-1          gtctggcctgcccaatgaggttttgaatggccgcagtatcctaactgtgctaaggtagca
16BB-2          gtctggcctgcccaatgaggttttgaatggccgcagtatcctaactgtgctaaggtagca
16YN-1          gtctggcctgcccaatgaggttttgaatggccgcagtatcctaactgtgctaaggtagca
16YN-2          gtctggcctgcccaatgaggttttgaatggccgcagtatcctaactgtgctaaggtagca
16FS-1          gtctggcctgcccaatgaggttttgaatggccgcagtatcctaactgtgctaaggtagca
16FS-2          gtctggcctgcccaatgaggttttgaatggccgcagtatcctaactgtgctaaggtagca
16LB            gtctggcctgcccaatgaggttttgaatggccgcagtatcctaactgtgctaaggtagca
16NM-1          gtctggcctgcccaatgaggttttgaatggccgcagtatcctaactgtgctaaggtagca
16NM-2          gtctggcctgcccaatgaggttttgaatggccgcagtatcctaactgtgctaaggtagca
16BH            gtctggcctgcccaatgaggttttgaatggccgcagtatcctaactgtgctaaggtagca
16HP-1          gtctggcctgcccaatgaggttttgaatggccgcagtatcctaactgtgctaaggtagca
16HP-2          gtctggcctgcccaatgaggttttgaatggccgcagtatcctaactgtgctaaggtagca
16HP-3          gtctggcctgcccaatgaggttttgaatggccgcagtatcctaactgtgctaaggtagca
                ************************************************************

16BB-1          taatcatttgtcttttaattgagggcttgtatgaaaggttggacaagatattaactttct
16BB-2          taatcatttgtcttttaattgagggcttgtatgaaaggttggacaagatattaactttct
16YN-1          taatcatttgtcttttaattgagggcttgtatgaaaggttggacaagatattaactttct
16YN-2          taatcatttgtcttttaattgagggcttgtatgaaaggttggacaagatattaactttct
16FS-1          taatcatttgtcttttaattgagggcttgtatgaaaggttggacaagatattaactttct
16FS-2          taatcatttgtcttttaattgagggcttgtatgaaaggttggacaagatattaactttct
16LB            taatcatttgtcttttaattgagggcttgtatgaaaggttggacaagatattaactttct
16NM-1          taatcatttgtcttttaattgagggcttgtatgaaaggttggacaagatattaactttct
16NM-2          taatcatttgtcttttaattgagggcttgtatgaaaggttggacaagatattaactttct
16BH            taatcatttgtcttttaattgagggcttgtatgaaaggttggacaagatattaactttct
16HP-1          taatcatttgtcttttaattgagggcttgtatgaaaggttggacaagatattaactttct
16HP-2          taatcatttgtcttttaattgagggcttgtatgaaaggttggacaagatattaactttct
16HP-3          taatcatttgtcttttaattgagggcttgtatgaaaggttggacaagatattaactttct
                ************************************************************

16BB-1          ttaatttaattttaagaacttaatttttaagttaaaaagcttaaatttttttgttggacg
16BB-2          ttaatttaattttaagaacttaatttttaagttaaaaagcttaaatttttttgttggacg
16YN-1          ttaatttaattttaagaacttaatttttaagttaaaaagcttaaatttttttgttggacg
16YN-2          ttaatttaattttaagaacttaatttttaagttaaaaagcttaaatttttttgttggacg
16FS-1          ttaatttaattttaagaacttaatttttaagttaaaaagcttaaatttttttgttggacg
16FS-2          ttaatttaattttaagaacttaatttttaagttaaaaagcttaaatttttttgttggacg
16LB            ttaatttaattttaagaacttaatttttaagttaaaaagcttaaatttttttgttggacg
16NM-1          ttaatttaattttaagaacttaatttttaagttaaaaagcttaaatttttttgttggacg
16NM-2          ttaatttaattttaagaacttaatttttaagttaaaaagcttaaatttttttgttggacg
16BH            ttaatttaattttaagaacttaatttttaagttaaaaagcttaaatttttttgttggacg
16HP-1          ttaatttaattttaagaacttaatttttaagttaaaaagcttaaatttttttgttggacg
16HP-2          ttaatttaattttaagaacttaatttttaagttaaaaagcttaaatttttttgttggacg
16HP-3          ttaatttaattttaagaacttaatttttaagttaaaaagcttaaatttttttgttggacg
                ************************************************************

16BB-1          agaagaccctatagatctttatagtttttagttttagtaatttagtggttaatttatttg
16BB-2          agaagaccctatagatctttatagtttttagttttagtaatttagtggttaatttatttg
16YN-1          agaagaccctatagatctttatagtttttagttttagtaatttagtggttaatttatttg
16YN-2          agaagaccctatagatctttatagtttttagttttagtaatttagtggttaatttatttg
16FS-1          agaagaccctatagatctttatagtttttagttttagtaatttagtggttaatttatttg
16FS-2          agaagaccctatagatctttatagtttttagttttagtaatttagtggttaatttatttg
16LB            agaagaccctatagatctttatagtttttagttttagtaatttagtggttaatttatttg
16NM-1          agaagaccctatagatctttatagtttttagttttagtaatttagtggttaatttatttg
16NM-2          agaagaccctatagatctttatagtttttagttttagtaatttagtggttaatttatttg
16BH            agaagaccctatagatctttatagtttttagttttagtaatttagtggttaatttatttg
16HP-1          agaagaccctatagatctttatagtttttagttttagtaatttagtggttaatttatttg
16HP-2          agaagaccctatagatctttatagtttttagttttagtaatttagtggttaatttatttg
16HP-3          agaagaccctatagatctttatagtttttagttttagtaatttagtggttaatttatttg
                ************************************************************

16BB-1          gagtgaaaaaattattttgttggggtgactgtagaattttatgaacttctatttttatat
16BB-2          gagtgaaaaaattattttgttggggtgactgtagaattttatgaacttctatttttatat
16YN-1          gagtgaaaaaattattttgttggggtgactgtagaattttatgaacttctatttttatat
16YN-2          gagtgaaaaaattattttgttggggtgactgtagaattttatgaacttctatttttatat
16FS-1          gagtgaaaaaattattttgttggggtgactgtagaattttatgaacttctatttttatat
```

```
16FS-2          gagtgaaaaaattattttgttggggtgactgtagaattttatgaacttctatttttatat
16LB            gagtgaaaaaattattttgttggggtgactgtagaattttatgaacttctatttttatat
16NM-1          gagtgaaaaaattattttgttggggtgactgtagaattttatgaacttctatttttatat
16NM-2          gagtgaaaaaattattttgttggggtgactgtagaattttatgaacttctatttttatat
16BH            gagtgaaaaaattattttgttggggtgactgtagaattttatgaacttctatttttatat
16HP-1          gagtgaaaaaattattttgttggggtgactgtagaattttatgaacttctatttttatat
16HP-2          gagtgaaaaaattattttgttggggtgactgtagaattttatgaacttctatttttatat
16HP-3          gagtgaaaaaattattttgttggggtgactgtagaattttatgaacttctatttttatat
                ************************************************************

16BB-1          ttcatatgttagtgtttttttgatccatatatttatgattagaagattaagttaccttag
16BB-2          ttcatatgttagtgtttttttgatccatatatttatgattagaagattaagttaccttag
16YN-1          ttcatatgttagtgtttttttgatccatatatttatgattagaagattaagttaccttag
16YN-2          ttcatatgttagtgtttttttgatccatatatttatgattagaagattaagttaccttag
16FS-1          ttcatatgttagtgtttttttgatccatatatttatgattagaagattaagttaccttag
16FS-2          ttcatatgttagtgtttttttgatccatatatttatgattagaagattaagttaccttag
16LB            ttcatatgttagtgtttttttgatccatatatttatgattagaagattaagttaccttag
16NM-1          ttcatatgttagtgtttttttgatccatatatttatgattagaagattaagttaccttag
16NM-2          ttcatatgttagtgtttttttgatccatatatttatgattagaagattaagttaccttag
16BH            ttcatatgttagtgtttttttgatccatatatttatgattagaagattaagttaccttag
16HP-1          ttcatatgttagtgtttttttgatccatatatttatgattagaagattaagttaccttag
16HP-2          ttcatatgttagtgtttttttgatccatatatttatgattagaagattaagttaccttag
16HP-3          ttcatatgttagtgtttttttgatccatatatttatgattagaagattaagttaccttag
                ************************************************************

16BB-1          ggataacagcgtaatttctttggagagttcatattgataaaggagtttgcgacctcgatg
16BB-2          ggataacagcgtaatttctttggagagttcatattgataaaggagtttgcgacctcgatg
16YN-1          ggataacagcgtaatttctttggagagttcatattgataaaggagtttgcgacctcgatg
16YN-2          ggataacagcgtaatttctttggagagttcatattgataaaggagtttgcgacctcgatg
16FS-1          ggataacagcgtaatttctttggagagttcatattgataaaggagtttgcgacctcgatg
16FS-2          ggataacagcgtaatttctttggagagttcatattgataaaggagtttgcgacctcgatg
16LB            ggataacagcgtaatttctttggagagttcatattgataaaggagtttgcgacctcgatg
16NM-1          ggataacagcgtaatttctttggagagttcatattgataaaggagtttgcgacctcgatg
16NM-2          ggataacagcgtaatttctttggagagttcatattgataaaggagtttgcgacctcgatg
16BH            ggataacagcgtaatttctttggagagttcatattgataaaggagtttgcgacctcgatg
16HP-1          ggataacagcgtaatttctttggagagttcatattgataaaggagtttgcgacctcgatg
16HP-2          ggataacagcgtaatttctttggagagttcatattgataaaggagtttgcgacctcgatg
16HP-3          ggataacagcgtaatttctttggagagttcatattgataaaggagtttgcgacctcgatg
                ************************************************************

16BB-1          ttggattaaaatgagttatggatgcagaagtctatatactgggtctgttcgacccttaat
16BB-2          ttggattaaaatgagttatggatgcagaagtctatatactgggtctgttcgacccttaat
16YN-1          ttggattaaaatgagttatggatgcagaagtctatatactgggtctgttcgacccttaat
16YN-2          ttggattaaaatgagttatggatgcagaagtctatatactgggtctgttcgacccttaat
16FS-1          ttggattaaaatgagttatggatgcagaagtctatatactgggtctgttcgacccttaat
16FS-2          ttggattaaaatgagttatggatgcagaagtctatatactgggtctgttcgacccttaat
16LB            ttggattaaaatgagttatggatgcagaagtctatatactgggtctgttcgacccttaat
16NM-1          ttggattaaaatgagttatggatgcagaagtctatatactgggtctgttcgacccttaat
16NM-2          ttggattaaaatgagttatggatgcagaagtctatatactgggtctgttcgacccttaat
16BH            ttggattaaaataagttatggatgcagaagtctatacactgggtctgttcgacccttaat
16HP-1          ttggattaaaataagttatggatgcagaagtctatacactgggtctgttcgacccttaat
16HP-2          ttggattaaaataagttatggatgcagaagtctatacactgggtctgttcgacccttaat
16HP-3          ttggattaaaataagttatggatgcagaagtctatacactgggtctgttcgacccttaat
                ************.***********************.***********************

16BB-1          tttttacatgatctgagtt
16BB-2          tttttacatgatctgagtt
16YN-1          tttttacatgatctgagtt
16YN-2          tttttacatgatctgagtt
16FS-1          tttttacatgatctgagtt
16FS-2          tttttacatgatctgagtt
16LB            tttttacatgatctgagtt
16NM-1          tttttacatgatctgagtt
16NM-2          tttttacatgatctgagtt
16BH            tttttacatgatctgagtt
16HP-1          tttttacatgatctgagtt
16HP-2          tttttacatgatctgagtt
16HP-3          tttttacatgatctgagtt
                *******************
```

The alignment of 28S rRNA gene of *Triatoma rubrofasciata* from Guangxi

```
28BB-1          ctgactgatcgctgccgaccggtgtaaataagcactgtccaaggaactgtttaccaacag
28BB-2          ctgactgatcgctgccgaccggtgtaaataagcactgtccaaggaactgtttaccaacag
28YN-1          ctgactgatcgctgccgaccggtgtaaataagcactgtccaaggaactgtttaccaacag
28YN-2          ctgactgatcgctgccgaccggtgtaaataagcactgtccaaggaactgtttaccaacag
28BH            ctgactgatcgctgccgaccggtgtaaataagcactgtccaaggaactgtttaccaacag
28FS-1          ctgactgatcgctgccgaccggtgtaaataagcactgtccaaggaactgtttaccaacag
28FS-2          ctgactgatcgctgccgaccggtgtaaataagcactgtccaaggaactgtttaccaacag
28LB            ctgactgatcgctgccgaccggtgtaaataagcactgtccaaggaactgtttaccaacag
28NM-1          ctgactgatcgctgccgaccggtgtaaataagcactgtccaaggaactgtttaccaacag
28NM-2          ctgactgatcgctgccgaccggtgtaaataagcactgtccaaggaactgtttaccaacag
28HP-1          ctgactgatcgctgccgaccggtgt-aataagcactgtccaaggaactgtttaccaacag
28HP-2          ctgactgatcgctgccgaccggtgt-aataagcactgtccaaggaactgtttaccaacag
28HP-3          ctgactgatcgctgccgaccggtgt-aataagcactgtccaaggaactgtttaccaacag
                ************************* **********************************

28BB-1          taggtggaatagttagacggactaaaaaatccgactgaaactaattttaaaccaacccac
28BB-2          taggtggaatagttagacggactaaaaaatccgactgaaactaattttaaaccaacccac
28YN-1          taggtggaatagttagacggactaaaaaatccgactgaaactaattttaaaccaacccac
28YN-2          taggtggaatagttagacggactaaaaaatccgactgaaactaattttaaaccaacccac
28BH            taggtggaatagttagacggactaaaaaatccgactgaaactaattttaaaccaacccac
28FS-1          taggtggaatagttagacggactaaaaaatccgactgaaactaattttaaaccaacccac
28FS-2          taggtggaatagttagacggactaaaaaatccgactgaaactaattttaaaccaacccac
28LB            taggtggaatagttagacggactaaaaaatccgactgaaactaattttaaaccaacccac
28NM-1          taggtggaatagttagacggactaaaaaatccgactgaaactaattttaaaccaacccac
28NM-2          taggtggaatagttagacggactaaaaaatccgactgaaactaattttaaaccaacccac
28HP-1          taggtggaatagttagacggactaaaaaatccgactgaaactaattttaaaccaacccac
28HP-2          taggtggaatagttagacggactaaaaaatccgactgaaactaattttaaaccaacccac
28HP-3          taggtggaatagttagacggactaaaaaatccgactgaaactaattttaaaccaacccac
                ************************************************************

28BB-1          ttgcaaacagccacaaaaaaaaggacgacactctccattatacaaaataataattttgta
28BB-2          ttgcaaacagccacaaaaaaaaggacgacactctccattatacaaaataataattttgta
28YN-1          ttgcaaacagccacaaaaaaaaggacgacactctccattatacaaaataataattttgta
28YN-2          ttgcaaacagccacaaaaaaaaggacgacactctccattatacaaaataataattttgta
28BH            ttgcaaacagccacaaaaaaaaggacgacactctccattatacaaaataataattttgta
28FS-1          ttgcaaacagccacaaaaaaaaggacgacactctccattatacaaaataataattttgta
28FS-2          ttgcaaacagccacaaaaaaaaggacgacactctccattatacaaaataataattttgta
28LB            ttgcaaacagccacaaaaaaaaggacgacactctccattatacaaaataataattttgta
28NM-1          ttgcaaacagccacaaaaaaaaggacgacactctccattatacaaaataataattttgta
28NM-2          ttgcaaacagccacaaaaaaaaggacgacactctccattatacaaaataataattttgta
28HP-1          ttgcaaacagccacaaaaaaaaggacgacactctccattatacaaaataataattttgta
28HP-2          ttgcaaacagccacaaaaaaaaggacgacactctccattatacaaaataataattttgta
28HP-3          ttgcaaacagccacaaaaaaaaggacgacactctccattatacaaaataataattttgta
                ************************************************************

28BB-1          caactttaagtggctcaacaccatcattcgttactgtcaaattatatccagtatacagtt
28BB-2          caactttaagtggctcaacaccatcattcgttactgtcaaattatatccagtatacagtt
28YN-1          caactttaagtggctcaacaccatcattcgttactgtcaaattatatccagtatacagtt
28YN-2          caactttaagtggctcaacaccatcattcgttactgtcaaattatatccagtatacagtt
28BH            caactttaagtggctcaacaccatcattcgttactgtcaaattatatccagtatacagtt
28FS-1          caactttaagtggctcaacaccatcattcgttactgtcaaattatatccagtatacagtt
28FS-2          caactttaagtggctcaacaccatcattcgttactgtcaaattatatccagtatacagtt
28LB            caactttaagtggctcaacaccatcattcgttactgtcaaattatatccagtatacagtt
28NM-1          caactttaagtggctcaacaccatcattcgttactgtcaaattatatccagtatacagtt
28NM-2          caactttaagtggctcaacaccatcattcgttactgtcaaattatatccagtatacagtt
28HP-1          caactttaagtggctcaacaccatcattcgttactgtcaaattatatccagtatacagtt
28HP-2          caactttaagtggctcaacaccatcattcgttactgtcaaattatatccagtatacagtt
28HP-3          caactttaagtggctcaacaccatcattcgttactgtcaaattatatccagtatacagtt
                ************************************************************

28BB-1          taacggctaaaactgttaaaaggcaaaatactttttacagtattccacactaaaaacagg
28BB-2          taacggctaaaactgttaaaaggcaaaatactttttacagtattccacactaaaaacagg
28YN-1          taacggctaaaactgttaaaaggcaaaatactttttacagtattccacactaaaaacagg
28YN-2          taacggctaaaactgttaaaaggcaaaatactttttacagtattccacactaaaaacagg
28BH            taacggctaaaactgttaaaaggcaaaatactttttacagtattccacactaaaaacagg
```

```
28FS-1          taacggctaaaactgttaaaaggcaaaatactttttacagtattccacactaaaaacagg
28FS-2          taacggctaaaactgttaaaaggcaaaatactttttacagtattccacactaaaaacagg
28LB            taacggctaaaactgttaaaaggcaaaatactttttacagtattccacactaaaaacagg
28NM-1          taacggctaaaactgttaaaaggcaaaatactttttacagtattccacactaaaaacagg
28NM-2          taacggctaaaactgttaaaaggcaaaatactttttacagtattccacactaaaaacagg
28HP-1          taacggctaaaactgttaaaaggcaaaatactttttacagtattccacactaaaaacagg
28HP-2          taacggctaaaactgttaaaaggcaaaatactttttacagtattccacactaaaaacagg
28HP-3          taacggctaaaactgttaaaaggcaaaatactttttacagtattccacactaaaaacagg
                ************************************************************

28BB-1          caaattagccatatacttaggatgttatttgacaggtcgcaacgtcctactaagggagaa
28BB-2          caaattagccatatacttaggatgttatttgacaggtcgcaacgtcctactaagggagaa
28YN-1          caaattagccatatacttaggatgttatttgacaggtcgcaacgtcctactaagggagaa
28YN-2          caaattagccatatacttaggatgttatttgacaggtcgcaacgtcctactaagggagaa
28BH            caaattagccatatacttaggatgttatttgacaggtcgcaacgtcctactaagggagaa
28FS-1          caaattagccatatacttaggatgttatttgacaggtcgcaacgtcctactaagggagaa
28FS-2          caaattagccatatacttaggatgttatttgacaggtcgcaacgtcctactaagggagaa
28LB            caaattagccatatacttaggatgttatttgacaggtcgcaacgtcctactaagggagaa
28NM-1          caaattagccatatacttaggatgttatttgacaggtcgcaacgtcctactaagggagaa
28NM-2          caaattagccatatacttaggatgttatttgacaggtcgcaacgtcctactaagggagaa
28HP-1          caaattagccatatacttaggatgttatttgacaggtcgcaacgtcctactaagggagaa
28HP-2          caaattagccatatacttaggatgttatttgacaggtcgcaacgtcctactaagggagaa
28HP-3          caaattagccatatacttaggatgttatttgacaggtcgcaacgtcctactaagggagaa
                ************************************************************

28BB-1          gtgcggtccaccattacccatccaatttaaatgcgagcgaccaaacccactaccacagtt
28BB-2          gtgcggtccaccattacccatccaatttaaatgcgagcgaccaaacccactaccacagtt
28YN-1          gtgcggtccaccattacccatccaatttaaatgcgagcgaccaaacccactaccacagtt
28YN-2          gtgcggtccaccattacccatccaatttaaatgcgagcgaccaaacccactaccacagtt
28BH            gtgcggtccaccattacccatccaatttaaatgcgagcgaccaaacccactaccacagtt
28FS-1          gtgcggtccaccattacccatccaatttaaatgcgagcgaccaaacccactaccacagtt
28FS-2          gtgcggtccaccattacccatccaatttaaatgcgagcgaccaaacccactaccacagtt
28LB            gtgcggtccaccattacccatccaatttaaatgcgagcgaccaaacccactaccacagtt
28NM-1          gtgcggtccaccattacccatccaatttaaatgcgagcgaccaaacccactaccacagtt
28NM-2          gtgcggtccaccattacccatccaatttaaatgcgagcgaccaaacccactaccacagtt
28HP-1          gtgcggtccaccattacccatccaatttaaatgcgagcgaccaaacccactaccacagtt
28HP-2          gtgcggtccaccattacccatccaatttaaatgcgagcgaccaaacccactaccacagtt
28HP-3          gtgcggtccaccattacccatccaatttaaatgcgagcgaccaaacccactaccacagtt
                ************************************************************

28BB-1          gttacactgaatagggcgagaggccatctcaaatcatttaaaagttgagcaactggtaag
28BB-2          gttacactgaatagggcgagaggccatctcaaatcatttaaaagttgagcaactggtaag
28YN-1          gttacactgaatagggcgagaggccatctcaaatcatttaaaagttgagcaactggtaag
28YN-2          gttacactgaatagggcgagaggccatctcaaatcatttaaaagttgagcaactggtaag
28BH            gttacactgaatagggcgagaggccatctcaaatcatttaaaagttgagcaactggtaag
28FS-1          gttacactgaatagggcgagaggccatctcaaatcatttaaaagttgagcaactggtaag
28FS-2          gttacactgaatagggcgagaggccatctcaaatcatttaaaagttgagcaactggtaag
28LB            gttacactgaatagggcgagaggccatctcaaatcatttaaaagttgagcaactggtaag
28NM-1          gttacactgaatagggcgagaggccatctcaaatcatttaaaagttgagcaactggtaag
28NM-2          gttacactgaatagggcgagaggccatctcaaatcatttaaaagttgagcaactggtaag
28HP-1          gttacactgaatagggcgagaggccatctcaaatcatttaaaagttgagcaactggtaag
28HP-2          gttacactgaatagggcgagaggccatctcaaatcatttaaaagttgagcaactggtaag
28HP-3          gttacactgaatagggcgagaggccatctcaaatcatttaaaagttgagcaactggtaag
                ************************************************************

28BB-1          acgtgaatctcccctttcggatattcaggtcttttccgtttacccctgagcggtttcacg
28BB-2          acgtgaatctcccctttcggatattcaggtcttttccgtttacccctgagcggtttcacg
28YN-1          acgtgaatctcccctttcggatattcaggtcttttccgtttacccctgagcggtttcacg
28YN-2          acgtgaatctcccctttcggatattcaggtcttttccgtttacccctgagcggtttcacg
28BH            acgtgaatctcccctttcggatattcaggtcttttccgtttacccctgagcggtttcacg
28FS-1          acgtgaatctcccctttcggatattcaggtcttttccgtttacccctgagcggtttcacg
28FS-2          acgtgaatctcccctttcggatattcaggtcttttccgtttacccctgagcggtttcacg
28LB            acgtgaatctcccctttcggatattcaggtcttttccgtttacccctgagcggtttcacg
28NM-1          acgtgaatctcccctttcggatattcaggtcttttccgtttacccctgagcggtttcacg
28NM-2          acgtgaatctcccctttcggatattcaggtcttttccgtttacccctgagcggtttcacg
28HP-1          acgtgaatctcccctttcggatattcaggtcttttccgtttacccctgagcggtttcacg
28HP-2          acgtgaatctcccctttcggatattcaggtcttttccgtttacccctgagcggtttcacg
28HP-3          acgtgaatctcccctttcggatattcaggtcttttccgtttacccctgagcggtttcacg
                ************************************************************
```

```
28BB-1          tactcttgaactctctcttcaaagttcttttcaactttccctcacggtacttgttcgcta
28BB-2          tactcttgaactctctcttcaaagttcttttcaactttccctcacggtacttgttcgcta
28YN-1          tactcttgaactctctcttcaaagttcttttcaactttccctcacggtacttgttcgcta
28YN-2          tactcttgaactctctcttcaaagttcttttcaactttccctcacggtacttgttcgcta
28BH            tactcttgaactctctcttcaaagttcttttcaactttccctcacggtacttgttcgcta
28FS-1          tactcttgaactctctcttcaaagttcttttcaactttccctcacggtacttgttcgcta
28FS-2          tactcttgaactctctcttcaaagttcttttcaactttccctcacggtacttgttcgcta
28LB            tactcttgaactctctcttcaaagttcttttcaactttccctcacggtacttgttcgcta
28NM-1          tactcttgaactctctcttcaaagttcttttcaactttccctcacggtacttgttcgcta
28NM-2          tactcttgaactctctcttcaaagttcttttcaactttccctcacggtacttgttcgcta
28HP-1          tactcttgaactctctcttcaaagttcttttcaactttccctcacggtacttgttcgcta
28HP-2          tactcttgaactctctcttcaaagttcttttcaactttccctcacggtacttgttcgcta
28HP-3          tactcttgaactctctcttcaaagttcttttcaactttccctcacggtacttgttcgcta
                ************************************************************

28BB-1          tcggtctcgtggttgtatttagccttagatggagtttaccacctgcttagggctgcacta
28BB-2          tcggtctcgtggttgtatttagccttagatggagtttaccacctgcttagggctgcacta
28YN-1          tcggtctcgtggttgtatttagccttagatggagtttaccacctgcttagggctgcacta
28YN-2          tcggtctcgtggttgtatttagccttagatggagtttaccacctgcttagggctgcacta
28BH            tcggtctcgtggttgtatttagccttagatggagtttaccacctgcttagggctgcacta
28FS-1          tcggtctcgtggttgtatttagccttagatggagtttaccacctgcttagggctgcacta
28FS-2          tcggtctcgtggttgtatttagccttagatggagtttaccacctgcttagggctgcacta
28LB            tcggtctcgtggttgtatttagccttagatggagtttaccacctgcttagggctgcacta
28NM-1          tcggtctcgtggttgtatttagccttagatggagtttaccacctgcttagggctgcacta
28NM-2          tcggtctcgtggttgtatttagccttagatggagtttaccacctgcttagggctgcacta
28HP-1          tcggtctcgtggttgtatttagccttagatggagtttaccacctgcttagggctgcacta
28HP-2          tcggtctcgtggttgtatttagccttagatggagtttaccacctgcttagggctgcacta
28HP-3          tcggtctcgtggttgtatttagccttagatggagtttaccacctgcttagggctgcacta
                ************************************************************

28BB-1          tcaagc
28BB-2          tcaagc
28YN-1          tcaagc
28YN-2          tcaagc
28BH            tcaagc
28FS-1          tcaagc
28FS-2          tcaagc
28LB            tcaagc
28NM-1          tcaagc
28NM-2          tcaagc
28HP-1          tcaagc
28HP-2          tcaagc
28HP-3          tcaagc
                ******
```

The alignment of Cytb gene of *Triatoma rubrofasciata* from Guangxi

```
BB-1            ttattatggatcatataaattattcatgacttgaagcattggggtgattttattattcat
BB-2            ttattatggatcatataaattattcatgacttgaagcattggggtgattttattattcat
YN-1            ttattatggatcatataaattattcatgacttgaagcattggggtgattttattattcat
YN-2            ttattatggatcatataaattattcatgacttgaagcattggggtgattttattattcat
FS-1            ttattatggatcatataaattattcatgacttgaagcattggggtgattttattattcat
FS-2            ttattatggatcatataaattattcatgacttgaagcattggggtgattttattattcat
LB              ttattatggatcatataaattattcatgacttgaagcattggggtgattttattattcat
NM-1            ttattatggatcatataaattattcatgacttgaagcattggggtgattttattattcat
NM-2            ttattatggatcatataaattattcatgacttgaagcattggggtgattttattattcat
BH              ttattatggatcatataaattattcatgacttgaagcattggggtgattttattattcat
HP-1            ttattatggatcatataaattattcatgacttgaagcattggggtgattttattattcat
HP-2            ttattatggatcatataaattattcatgacttgaagcattggggtgattttattattcat
HP-3            ttattatggatcatataaattattcatgacttgaagcattggggtgattttattattcat
                ************************************************************
```

```
BB-1            aattataggaactgcatttttagggtatgtcttaccctgaggacaaatatccttatgagg
BB-2            aattataggaactgcatttttagggtatgtcttaccctgaggacaaatatccttatgagg
YN-1            aattataggaactgcatttttagggtatgtcttaccctgaggacaaatatccttatgagg
YN-2            aattataggaactgcatttttagggtatgtcttaccctgaggacaaatatccttatgagg
FS-1            aattataggaactgcatttttagggtatgtcttaccctgaggacaaatatccttatgagg
FS-2            aattataggaactgcatttttagggtatgtcttaccctgaggacaaatatccttatgagg
LB              aattataggaactgcatttttagggtatgtcttaccctgaggacaaatatccttatgagg
NM-1            aattataggaactgcatttttagggtatgtcttaccctgaggacaaatatccttatgagg
NM-2            aattataggaactgcatttttagggtatgtcttaccctgaggacaaatatccttatgagg
BH              aattataggaactgcatttttagggtatgtcttaccctgaggacaaatatccttatgagg
HP-1            aattataggaactgcatttttagggtatgtcttaccctgaggacaaatatccttatgagg
HP-2            aattataggaactgcatttttagggtatgtcttaccctgaggacaaatatccttatgagg
HP-3            aattataggaactgcatttttagggtatgtcttaccctgaggacaaatatccttatgagg
                ************************************************************

BB-1            agcaacagttattactaacttgttatctgctatcccgtacttaggaaatgacttagtcat
BB-2            agcaacagttattactaacttgttatctgctatcccgtacttaggaaatgacttagtcat
YN-1            agcaacagttattactaacttgttatctgctatcccgtacttaggaaatgacttagtcat
YN-2            agcaacagttattactaacttgttatctgctatcccgtacttaggaaatgacttagtcat
FS-1            agcaacagttattactaacttgttatctgctatcccgtacttaggaaatgacttagtcat
FS-2            agcaacagttattactaacttgttatctgctatcccgtacttaggaaatgacttagtcat
LB              agcaacagttattactaacttgttatctgctatcccgtacttaggaaatgacttagtcat
NM-1            agcaacagttattactaacttgttatctgctatcccgtacttaggaaatgacttagtcat
NM-2            agcaacagttattactaacttgttatctgctatcccgtacttaggaaatgacttagtcat
BH              agcaacagttattactaacttgttatctgctatcccatacttaggaaatgatttagtcat
HP-1            agcaacagttattactaacttgttatctgctatcccatacttaggaaatgatttagtcat
HP-2            agcaacagttattactaacttgttatctgctatcccatacttaggaaatgatttagtcat
HP-3            agcaacagttattactaacttgttatctgctatcccatacttaggaaatgatttagtcat
                ************************************.**************.********

BB-1            atgattatgagggggattctcagtagataacgctactttaactcgattctttgccctaca
BB-2            atgattatgagggggattctcagtagataacgctactttaactcgattctttgccctaca
YN-1            atgattatgagggggattctcagtagataacgctactttaactcgattctttgccctaca
YN-2            atgattatgagggggattctcagtagataacgctactttaactcgattctttgccctaca
FS-1            atgattatgagggggattctcagtagataacgctactttaactcgattctttgccctaca
FS-2            atgattatgagggggattctcagtagataacgctactttaactcgattctttgccctaca
LB              atgattatgagggggattctcagtagataacgctactttaactcgattctttgccctaca
NM-1            atgattatgagggggattctcagtagataacgccactttaactcgattctttgccctaca
NM-2            atgattatgagggggattctcagtagataacgccactttaactcgattctttgccctaca
BH              atgattatgagggggattctcagtagataacgctactttaactcgattctttgccctaca
HP-1            atgattatgagggggattctcagtagataacgctactttaactcgattctttgccctaca
HP-2            atgattatgagggggattctcagtagataacgctactttaactcgattctttgccctaca
HP-3            atgattatgagggggattctcagtagataacgctactttaactcgattctttgccctaca
                *********************************.**************************

BB-1            tttcctttttaccattcattattgcagcattagtattaatccatttactctttctccatca
BB-2            tttcctttttaccattcattattgcagcattagtattaatccatttactctttctccatca
YN-1            tttcctttttaccattcattattgcagcattagtattaatccatttactctttctccatca
YN-2            tttcctttttaccattcattattgcagcattagtattaatccatttactctttctccatca
FS-1            tttcctttttaccattcattattgcagcattagtattaatccatttactctttctccatca
FS-2            tttcctttttaccattcattattgcagcattagtattaatccatttactctttctccatca
LB              tttcctttttaccattcattattgcagcattagtattaatccatttactctttctccatca
NM-1            tttcctttttaccattcattattgcagcattagtattaatccatttactctttctccatca
NM-2            tttcctttttaccattcattattgcagcattagtattaatccatttactctttctccatca
BH              tttcctttttaccattcattattgcagcattagtattaatccatttactctttctccatca
HP-1            tttcctttttaccattcattattgcagcattagtattaatccatttactctttctccatca
HP-2            tttcctttttaccattcattattgcagcattagtattaatccatttactctttctccatca
HP-3            tttcctttttaccattcattattgcagcattagtattaatccatttactctttctccatca
                ************************************************************

BB-1            aacaggatctaataatccattaggattaaatagaaattttgataaaatcccatttcaccc
BB-2            aacaggatctaataatccattaggattaaatagaaattttgataaaatcccatttcaccc
YN-1            aacaggatctaataatccattaggattaaatagaaattttgataaaatcccatttcaccc
YN-2            aacaggatctaataatccattaggattaaatagaaattttgataaaatcccatttcaccc
FS-1            aacaggatctaataatccattaggattaaatagaaattttgataaaatcccatttcaccc
FS-2            aacaggatctaataatccattaggattaaatagaaattttgataaaatcccatttcaccc
LB              aacaggatctaataatccattaggattaaatagaaattttgataaaatcccatttcaccc
NM-1            aacaggatctaataatccattaggattaaatagaaattttgataaaatcccatttcaccc
```

```
NM-2            aacaggatctaataatccattaggattaaatagaaattttgataaaatcccatttcaccc
BH              aacaggatctaataatccattaggattaaatagaaattttgataaaatcccatttcaccc
HP-1            aacaggatctaataatccattaggattaaatagaaattttgataaaatcccatttcaccc
HP-2            aacaggatctaataatccattaggattaaatagaaattttgataaaatcccatttcaccc
HP-3            aacaggatctaataatccattaggattaaatagaaattttgataaaatcccatttcaccc
                ************************************************************

BB-1            atatttctctattaaagaccttataggagtatcaataaccccttatattctttatcctact
BB-2            atatttctctattaaagaccttataggagtatcaataaccccttatattctttatcctact
YN-1            atatttctctattaaagaccttataggagtatcaataaccccttatattctttatcctact
YN-2            atatttctctattaaagaccttataggagtatcaataaccccttatattctttatcctact
FS-1            atatttctctattaaagaccttataggagtatcaataaccccttatattctttatcctact
FS-2            atatttctctattaaagaccttataggagtatcaataaccccttatattctttatcctact
LB              atatttctctattaaagaccttataggagtatcaataaccccttatattctttatcctact
NM-1            atatttctctattaaagaccttataggagtatcaataaccccttatattctttatcctact
NM-2            atatttctctattaaagaccttataggagtatcaataaccccttatattctttatcctact
BH              atatttctctattaaggaccttataggagtatcaataaccccttatattctttatcctact
HP-1            atatttctctattaaggaccttataggagtatcaataaccccttatattctttatcctact
HP-2            atatttctctattaaggaccttataggagtatcaataaccccttatattctttatcctact
HP-3            atatttctctattaaggaccttataggagtatcaataaccccttatattctttatcctact
                ***************.********************************************

BB-1            aaacctttgagaacctcgattattgggagaccctgaaaactttatcccagccaacccatt
BB-2            aaacctttgagaacctcgattattgggagaccctgaaaactttatcccagccaacccatt
YN-1            aaacctttgagaacctcgattattgggagaccctgaaaactttatcccagccaacccatt
YN-2            aaacctttgagaacctcgattattgggagaccctgaaaactttatcccagccaacccatt
FS-1            aaacctttgagaacctcgattattgggagaccctgaaaactttatcccagccaacccatt
FS-2            aaacctttgagaacctcgattattgggagaccctgaaaactttatcccagccaacccatt
LB              aaacctttgagaacctcgattattgggagaccctgaaaactttatcccagccaacccatt
NM-1            aaacctttgagaacctcgattattgggagaccctgaaaactttatcccagccaacccatt
NM-2            aaacctttgagaacctcgattattgggagaccctgaaaactttatcccagccaacccatt
BH              aaacctttgagaacctcgattattgggagaccctgaaaactttatcccagccaacccatt
HP-1            aaacctttgagaacctcgattattgggagaccctgaaaactttatcccagccaacccatt
HP-2            aaacctttgagaacctcgattattgggagaccctgaaaactttatcccagccaacccatt
HP-3            aaacctttgagaacctcgattattgggagaccctgaaaactttatcccagccaacccatt
                ************************************************************

BB-1            agttaccccggttcatatccaaccagaatggtattttctatttgcatacgcaattctacg
BB-2            agttaccccggttcatatccaaccagaatggtattttctatttgcatacgcaattctacg
YN-1            agttaccccggttcatatccaaccagaatggtattttctatttgcatacgcaattctacg
YN-2            agttaccccggttcatatccaaccagaatggtattttctatttgcatacgcaattctacg
FS-1            agttaccccggttcatatccaaccagaatggtattttctatttgcatacgcaattctacg
FS-2            agttaccccggttcatatccaaccagaatggtattttctatttgcatacgcaattctacg
LB              agttaccccggttcatatccaaccagaatggtattttctatttgcatacgcaattctacg
NM-1            agttaccccggttcatatccaaccagaatggtattttctatttgcatacgcaattctacg
NM-2            agttaccccggttcatatccaaccagaatggtattttctatttgcatacgcaattctacg
BH              agttaccccggttcatatccaaccagaatggtattttctatttgcatacgcaattctacg
HP-1            agttaccccggttcatatccaaccagaatggtattttctatttgcatacgcaattctacg
HP-2            agttaccccggttcatatccaaccagaatggtattttctatttgcatacgcaattctacg
HP-3            agttaccccggttcatatccaaccagaatggtattttctatttgcatacgcaattctacg
                ************************************************************

BB-1            atctattcctaataaactaggaggggtaattgcaatagtttcttccatcataattattat
BB-2            atctattcctaataaactaggaggggtaattgcaatagtttcttccatcataattattat
YN-1            atctattcctaataaactaggaggggtaattgcaatagtttcttccatcataattattat
YN-2            atctattcctaataaactaggaggggtaattgcaatagtttcttccatcataattattat
FS-1            atctattcctaataaactaggaggggtaattgcaatagtttcttccatcataattattat
FS-2            atctattcctaataaactaggaggggtaattgcaatagtttcttccatcataattattat
LB              atctattcctaataaactaggaggggtaattgcaatagtttcttccatcataattattat
NM-1            atctattcctaataaactaggaggggtaattgcaatagtttcttccatcataattattat
NM-2            atctattcctaataaactaggaggggtaattgcaatagtttcttccatcataattattat
BH              atctattcctaataaactaggaggggtaattgcaatagtttcttccatcgtaattattat
HP-1            atctattcctaataaactaggaggggtaattgcaatagtttcttccatcgtaattattat
HP-2            atctattcctaataaactaggaggggtaattgcaatagtttcttccatcgtaattattat
HP-3            atctattcctaataaactaggaggggtaattgcaatagtttcttccatcgtaattattat
                *************************************************.**********

BB-1            aattctaccaattaccaacaaaagaaaatttcaaggaatttctttttatccaataaatca
BB-2            aattctaccaattaccaacaaaagaaaatttcaaggaatttctttttatccaataaatca
```

```
YN-1            aattctaccaattaccaacaaaagaaaatttcaaggaatttctttttatccaataaatca
YN-2            aattctaccaattaccaacaaaagaaaatttcaaggaatttctttttatccaataaatca
FS-1            aattctaccaattaccaacaaaagaaaatttcaaggaatttctttttatccaataaatca
FS-2            aattctaccaattaccaacaaaagaaaatttcaaggaatttctttttatccaataaatca
LB              aattctaccaattaccaacaaaagaaaatttcaaggaatttctttttatccaataaatca
NM-1            aattctaccaattaccaacaaaagaaaatttcaaggaatttctttttatccaataaatca
NM-2            aattctaccaattaccaacaaaagaaaatttcaaggaatttctttttatccaataaatca
BH              aattctaccaattaccaacaaaagaaaatttcaaggaatttcctttttatccaataaatca
HP-1            aattctaccaattaccaacaaaagaaaatttcaaggaatttcctttttatccaataaatca
HP-2            aattctaccaattaccaacaaaagaaaatttcaaggaatttcctttttatccaataaatca
HP-3            aattctaccaattaccaacaaaagaaaatttcaaggaatttcctttttatccaataaatca
                *****************************************.******************

BB-1            agtatct
BB-2            agtatct
YN-1            agtatct
YN-2            agtatct
FS-1            agtatct
FS-2            agtatct
LB              agtatct
NM-1            agtatct
NM-2            agtatct
BH              agtatct
HP-1            agtatct
HP-2            agtatct
HP-3            agtatct
                *******
```

Note: The 16S rRNA, 28S rRNA and cytochrome b (CytB) sequences of 13 *Triatoma rubrofasciata from Guangxi were* aligned by MAFFT (https://mafft.cbrc.jp/alignment/server/).
